# World Kidney Day 2026 in Africa: bridging environmental sustainability with the realities of kidney care

**DOI:** 10.1080/0886022X.2026.2700887

**Published:** 2026-09-16

**Authors:** Victorine Bandolo Nzana, Grace Kansiime, Zybia Barday, Yasminatou Wendkuuni Biikinga, Mohammed Abdel Gawad, Bianca Davidson, Yannick Mayamba Nlandu

**Affiliations:** ^a^ Medicine and Biomedical Sciences, University of Yaounde I, Cameroon; ^b^ Department of Medicine, Mbarara University of Science and Technology, Mbarara, Uganda; ^c^ Division of Nephrology and Hypertension, University of Cape Town, Cape Town, South Africa; ^d^ Department of Nephrology and hemodialysis, Bogodogo University Hospital Centre, Burkina Faso; ^e^ Nephrology Unit, Department of Internal Medicine, School of Medicine, Newgiza University (NGU), Giza, Egypt; ^f^ Department of Internal Medicine, Kinshasa Hospital University, Kinshasa, University of Kinshasa, Democratic Republic of the Congo

## Introduction

Chronic kidney disease (CKD) is recognized as an important public health concern due to its significant prevalence, mortality rate and the substantial financial burden it places on patients and health care systems.

The prevalence of CKD in Africa varies according to the region, population, healthcare system and existence of a national program for early detection. Overall, the prevalence of CKD in Africa is approximately 16%, with sub-Saharan regions bearing the highest prevalence rates [1,2]. However, depending on the population studied, high prevalence rates have been detected amongst high risk groups such as those with hypertension (35.6%), diabetes (32.6%) and HIV infection (27.3%) [2,3]. In comparison to other regions across the world, Africa seems to have a higher prevalence of CKD [4]. Moreover, this prevalence may be underestimated due to limited access to screening and diagnostic services, weak health systems, and poor disease surveillance in many African settings, which contribute to delayed detection and under-recognition of early-stage CKD [5–7].

World Kidney Day (WKD), established by the International Society of Nephrology and the International Federation of Kidney Foundations, serves as a global platform to raise awareness and drive action on kidney health [8]. Over the years, its themes have evolved from a focus on awareness and prevention to addressing equity, access to care, and health system resilience. Increasingly, the global kidney community has also recognized the influence of environmental and climate-related factors on kidney health [9,10]. The 2026 theme, “Kidney Health for All: Caring for People, Protecting the Planet,” reflects this shift by explicitly linking kidney care with environmental sustainability. This is particularly relevant for regions such as Africa, where the dual burden of kidney disease and climate vulnerability is substantial and often compounded by fragile health systems.

## The burden and determinants of kidney disease in Africa

In adults, the leading causes of CKD are reported to be hypertensive nephrosclerosis followed by diabetes and chronic glomerulonephritis [11]. However, a significant proportion of patients present with CKD of unknown or miscellaneous causes [12]. In Africa, the spectrum of CKD may be impacted by other infectious diseases such as schistosomiasis, tuberculosis, malaria and hepatitis B and C [13]. Additionally, genetic factors unique to populations of African ancestry, including sickle cell disease and APOL1 risk alleles, play an important role in susceptibility to CKD and its progression [14,15]. Poverty, a well-established risk factor for CKD is highly prevalent in some African settings and is compounded by overcrowding, inadequate supply of safe water, poor sanitation and pervasive environmental pollution [3]. The impact of severe or recurrent acute kidney injury (AKI), especially pregnancy-related AKI, on the development and progression of CKD, is another growing concern across Africa [16].

CKD is associated with rising morbidity and mortality, contributing to total deaths and disabilities worldwide. From 1990 to 2019, the number of deaths due to CKD doubled, ranking it as the 10^th^ leading cause of death worldwide. Estimates suggest that it will become the 5^th^ leading cause of death by 2040 [17,18]. The absolute number of Disability-Adjusted Life Years (DALYs) has more than doubled since 1990. These statistics demonstrate the negative impact of CKD on Africa’s economic growth, given that the population affected by CKD is relatively young and of working age. The lack of universal health coverage and specific nephrology care in many African countries often leads to the late presentation and diagnosis of advanced CKD [19]. The high cost and disparities in the distribution of dialysis centers across African countries limit access to kidney replacement therapy (KRT) [20]. In many circumstances, patients are unable to initiate or continue dialysis as they are required to pay out of pocket, which renders treatment unaffordable for the majority.

## Kidney disease and the environment: a bidirectional relationship

As WKD evolved, it increasingly recognized the disproportionate impact of CKD in low‑resource settings and the role of socioeconomic and environmental determinants in shaping kidney health. This progression has laid a natural foundation for the 2026 theme, “Kidney Health for All: Caring for People, Protecting the Planet,” which brings together the dual priorities of improving kidney care while addressing environmental and societal factors.

In light of climate change, there has been increasing global focus to environmental risk factors for kidney disease [10]. This is particularly relevant for Africa, where populations may be disproportionately exposed, although region-specific evidence remains limited [21]. Environmental and climate factors are increasingly recognized as kidney risk multipliers. Heat exposure, recurrent dehydration, water and air pollution, changing patterns of infections, and extreme weather events contribute to acute kidney injury and accelerate CKD progression [10,22]. Extreme weather events and climate-related disasters particularly floods, hurricanes, and storms can severely disrupt access to kidney care by damaging health facilities, interrupting electricity and water supplies, and limiting transportation, leading to missed or delayed dialysis sessions, reduced access to immunosuppressive therapy in kidney transplant recipients; and resulting in worsened clinical outcomes [23–25].

**Figure 1.**
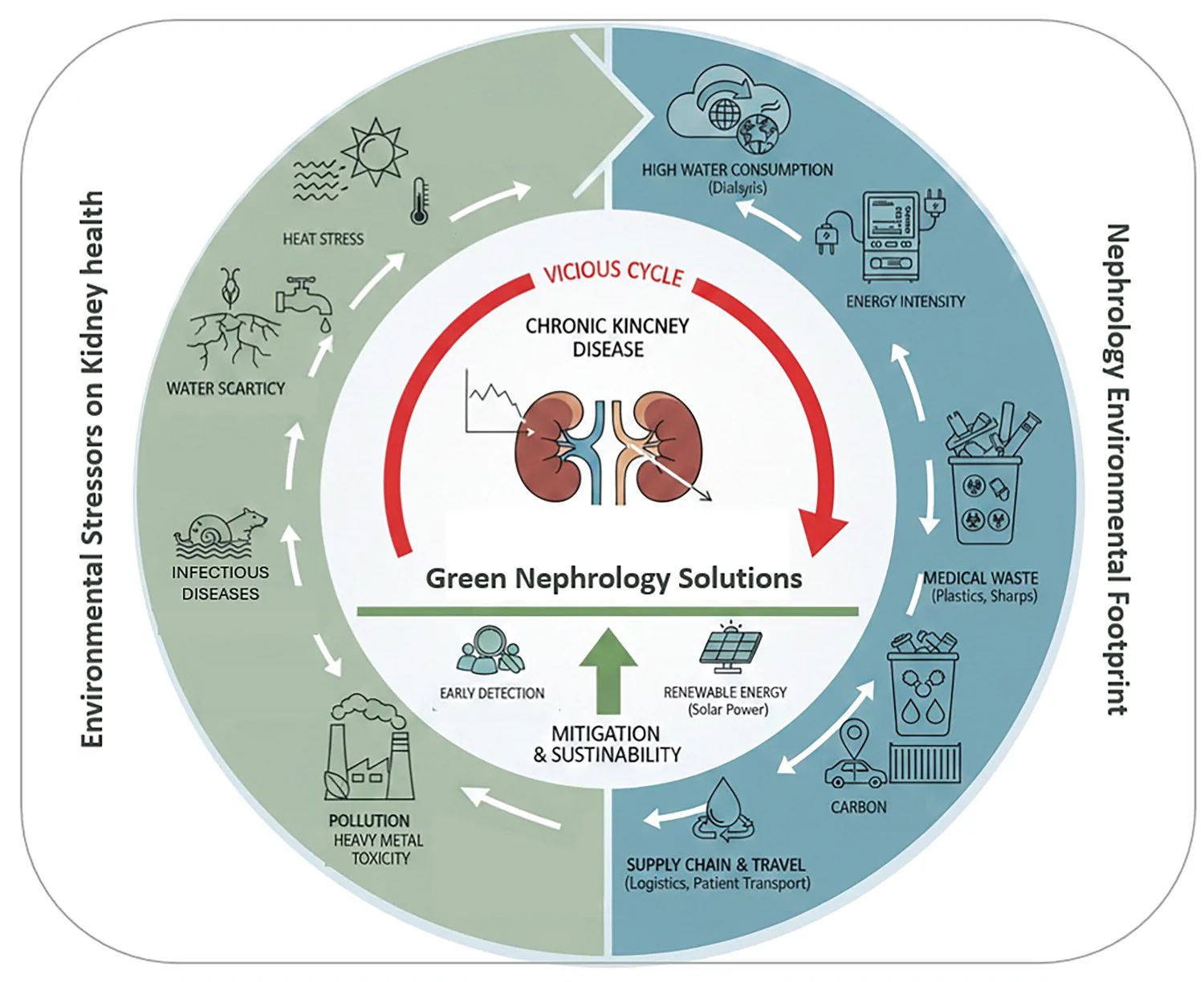
The bidirectional relationship between kidney and environment.

While there is compelling evidence that climate change adversely affects kidney health through rising temperatures, heat stress, water scarcity, and environmental pollution, the treatment of kidney disease itself exerts a considerable environmental impact. Kidney care, particularly dialysis, leaves a long-lasting environmental footprint and, paradoxically, contributes to the very climate crisis that amplifies kidney disease burden (Figure 1). This bidirectional relationship has been described as a self-perpetuating loop with no clear endpoint, in which climate change worsens kidney health, and kidney care further accelerates environmental degradation [26]. Dialysis, with hemodialysis at the top of the list, is particularly resource-intensive. It relies heavily on large volumes of water and energy, and it generates significant amounts of single-use plastic and medical waste. This contributes to greenhouse gas emissions and environmental pollution. Globally, an estimated 2–3 million people receive dialysis, and this number is projected to double by 2030 [27,28]. While dialysis is a lifesaving treatment, it comes at a high environmental cost. A single hemodialysis treatment session uses an average of 490 L of water, exceeding 150% of the average daily water use of a typical American household [29,30]. In addition, a hemodialysis facility is estimated to consume enough electricity to power more than 100 homes [31–33]. Moreover, hemodialysis is estimated to generate more than 2 million tons of waste annually, with a significant portion being potentially hazardous [28,30,33]. The transition from conventional hemodialysis to hemodiafiltration, while associated with improved quality of life and survival, is accompanied by continued and substantial environmental impact.

The environmental burden of kidney disease extends well beyond dialysis. Life-cycle assessment studies demonstrate that care across all stages of chronic kidney disease (CKD) generates substantial greenhouse gas emissions, with the per-patient carbon footprint increasing progressively as kidney function declines. Importantly, for patients in early to moderate CKD (stages 1–4), emissions are driven predominantly by hospitalizations, outpatient visits, and patient and staff travel, whereas dialysis modality becomes the dominant contributor only in advanced disease [33,34]. Hospital admissions, particularly, emergency and inpatient care, represent the largest source of emissions across most CKD stages, underscoring the environmental co-benefits of early detection, prevention of disease progression, and avoidance of potentially preventable hospitalizations [33,35].

These concerns have informed initiatives such as the ISN’s Green-K program, which emphasizes which promotes environmentally sustainable kidney care by reducing the ecological footprint of nephrology services while addressing the impact of climate change on kidney health [36]. Despite these efforts, implementation of environmentally sustainable practices remains slow globally and is particularly constrained in low- and middle-income countries, where limited resources and competing health priorities restrict the ability to adopt “green” technologies [36–39].

## Kidney care and kidney replacement therapy in Africa: realities, gaps, and tradeoffs

Despite the high burden of chronic kidney disease and late presentation, access to kidney care remains extremely limited across many African settings. Health systems face substantial constraints, including a limited nephrology workforce, inadequate infrastructure, and restricted availability of diagnostic and treatment services. Across the region, the density of nephrologists remains among the lowest globally, estimated at approximately 1.12 per million population, compared to a global median of over 11 per million. This shortage extends to other key cadres, including dialysis nurses, transplant surgeons, and interventional specialists, with more than half of countries reporting workforce gaps across these areas [40]. These gaps are further compounded by geographic disparities, with services concentrated in urban centers, leaving large rural populations underserved [41].

For individuals with advanced chronic kidney disease, kidney replacement therapy is often the only life-sustaining option; however, access to these therapies remains extremely limited across many African settings, shaping the realities of care delivery across the continent. Hemodialysis remains the most widely available and utilized modality of kidney replacement therapy across the continent. Although in-centre hemodialysis is present in most African countries, access remains highly variable, with significant disparities in availability, affordability, and utilization between and within countries [42]. While there is growing global emphasis on environmentally sustainable kidney care, efforts to implement “green nephrology” practices within hemodialysis such as water conservation, energy efficiency, and waste management, remain limited across African settings. Although some countries such as Morocco have explored strategies such as reuse of dialysis-related water and improved resource management, these are not widely implemented [42–44].

The ecological transition, therefore, remains slow even in high-income countries, while in low and middle-income countries, the implementation is frequently constrained by infrastructural, financial, and regulatory challenges hence limiting the ability to cope with the planet’s urgent needs.

As a result, despite its substantial environmental footprint, hemodialysis continues to serve as the default modality of kidney replacement therapy in many African countries, reflecting the prioritization of access to life-saving treatment over environmental sustainability.

Across Africa, peritoneal dialysis (PD), which offers potential environmental advantages through lower water and energy requirements, is not well-established and remains underutilized, with limited availability, variable access, and persistently low uptake. This is due to challenges related to supply chains, cost and availability of dialysis fluids, housing conditions, and risk of complications such as peritonitis [45,46]. In some countries such as South Africa, peritoneal dialysis (PD) is well established and has been promoted as a home-based therapy that may reduce transport-related emissions and infrastructure burden compared with in-center HD [46]. However, it still faces a lot of setbacks such as peritonitis [46].

In other settings, implementation has been inconsistent. For example, although PD was introduced in Egypt in 1972, its development has been marked by intermittent initiatives, limiting its widespread adoption [47].

In many other African countries including Uganda, Ethiopia, Ghana and Cameroon, peritoneal dialysis services are largely restricted to pediatric acute kidney injury care, with very limited availability for adults and/or chronic care [48–50].

## WKD 2026 theme: a call to action for Africa

The 2026 theme represents a natural and necessary progression from the previous themes. Africa carries a high and often under-recognized burden of CKD, with prevalence estimates exceeding global averages in high-risk populations. At the same time, the continent is uniquely vulnerable to climate change, heat stress, dehydration, environmental toxins, and fragile health systems; all of which increase the risk of acute and chronic kidney disease. The growing emphasis on environmentally sustainable kidney care presents important opportunities but also significant challenges in African settings, where access to basic kidney services remains limited. For countries already grappling with constrained resources and climate vulnerability, sustainable and preventive approaches to kidney care are both a health and environmental imperative.

## An African agenda for caring for people and the planet

For Africa and other low-and middle-income countries (LMICs), the 2026 theme demands action across the continuum of care.

### Prioritize integration of CKD screening into existing primary care and chronic disease platforms

In many African settings, CKD screening such as urine dipstick testing and serum creatinine measurement including the use of point-of-care creatinine testing, can be embedding within established hypertension, diabetes, and HIV clinics. The use of simplified diagnostic algorithms and equations to interpret kidney function can further support decision-making at primary care level, particularly in settings with limited specialist access [51–53]. Leveraging existing service delivery platforms allows for early identification of kidney disease without requiring parallel infrastructure, making this a practical and scalable approach in resource-constrained settings. This approach not only improves early diagnosis but also reduces progression to kidney failure, thereby decreasing reliance on resource-intensive therapies such as dialysis.

### Strengthen control of key modifiable risk factors through standardized, low-cost management pathways

Improving the detection and consistent management of hypertension, diabetes, and infections such as HIV should be prioritized across all levels of care. This can be achieved through simplified treatment protocols, task-shifting to non-physician healthcare workers, and integration into existing primary care programs. Ensuring continuity of care through follow-up systems and patient counseling is critical to slowing CKD progression and reducing preventable kidney failure.

### Improve access to essential medicines for CKD risk reduction and progression control

Ensuring equitable and consistent availability and affordability of essential medicines particularly antihypertensives, such as renin–angiotensin system inhibitors, and diabetes therapies is critical to slowing CKD progression in African settings [54–57]. Current individual national efforts can be strengthened through regional and continental pooled procurement mechanisms, such as the emerging African Pooled Procurement Mechanism, an African Union–led initiative to leverage collective bargaining power to negotiate lower prices and improve the availability of essential medicines across countries [58]. Expanding access beyond tertiary centers to the primary care level is essential to ensure that patients diagnosed early can receive appropriate treatment. In addition, subsidized access to essential medicines through public financing, insurance schemes, or targeted subsidy programs is critical to reducing out-of-pocket costs, which remain a major barrier to treatment adherence in many settings [58].

### Optimize existing dialysis services through efficient and context-appropriate resource use

While access to dialysis remains limited across many African countries, efforts should focus on improving the efficiency and sustainability of existing services rather than expansion alone. Practical, low-cost measures may include reuse of reverse osmosis reject water for non-clinical purposes, improved waste segregation to reduce unnecessary disposal of non-hazardous materials, and rational water use during dialysis delivery. Optimizing dialysis schedules and resource utilization within existing units can further improve efficiency without additional infrastructure.

In addition, basic energy-saving approaches such as reducing reliance on generators where possible or integrating alternative energy sources may offer incremental benefits. These context-appropriate interventions allow for gradual incorporation of environmentally sustainable practices without compromising access to life-saving treatment, aligning sustainability efforts with real-world health system constraints. It must nevertheless be acknowledged that, although this is theoretically possible, the implementation of these environmentally friendly practices remains slow on a global scale and faces considerable obstacles, particularly in low- and middle-income countries.

### Strengthen context-appropriate kidney replacement therapy options through incremental and locally feasible approaches

As Africa works to improve access to kidney replacement therapy, efforts should focus on strengthening modalities that are feasible within existing system constraints.

For peritoneal dialysis, this includes improving supply chains, ensuring consistent availability of dialysis fluids, and exploring opportunities for local or regional production to reduce costs and dependence on imports. Strengthening training for catheter insertion and prevention of complications such as peritonitis is also essential to support safe scale-up.

One of the major challenges for PD across Africa is its import-dependence for PD fluids. International and regional collaboration offers an important opportunity to strengthen access to peritoneal dialysis. Emerging continental initiatives, such as pooled procurement mechanisms, could enable countries to aggregate demand, improve bargaining power, and enhance the availability and affordability of dialysis consumables, including PD fluids. In this context, professional networks such as the African Renal Association (AFRAN) continue to play a key role in advocacy, coordination, and capacity building to support the development of context-appropriate PD programs across the region.

For kidney transplantation, incremental expansion of existing programs through regional collaboration, capacity building, and partnerships can improve access over time. From November 2021 to date, Cameroon has done a total 19 successful living donor kidney transplantations, and with the support of international partners, Senegal has successfully performed their first transplants in November 2023, while Uganda has carried out 14 living donor transplants since December 2024 [59]. These successes demonstrate that kidney transplantation is increasingly becoming a key focus of sustainable nephrology in Africa. They also highlight the positive impact of partnerships in gradually expanding access to care. Strengthening systems for donor evaluation, surgical capacity, and long-term access to immunosuppressive therapy is critical to ensuring sustainability. These approaches emphasize gradual system strengthening rather than rapid expansion, aligning with the realities of resource-constrained settings while improving access to more effective and potentially sustainable therapies.

In addition, resilience must be built into kidney care systems. Emergency preparedness plans, supply chain strengthening, and regional collaboration are essential to protect vulnerable patients during climate-related disasters and health system readiness [60–62].

## Conclusion

As World Kidney Day enters its third decade, the 2026 theme offers a call to action, a powerful synthesis of past advocacy messages. Caring for people with kidney disease and protecting the planet are not competing priorities; they are inseparable. For Africa, placing kidney health within the broader agenda of universal health coverage, climate adaptation and sustainable healthcare is no longer optional. It is the only viable path to reducing the growing burden of kidney disease while safeguarding the environments on which current and future generations depend.

## Data Availability

No new data was generated or analyzed in this comment.
